# HBx Sensitizes Cells to Oxidative Stress-induced Apoptosis by Accelerating the Loss of Mcl-1 Protein via Caspase-3 Cascade

**DOI:** 10.1186/1476-4598-10-43

**Published:** 2011-04-20

**Authors:** Liang Hu, Lei Chen, GuangZhen Yang, Liang Li, HanYong Sun, YanXin Chang, QianQian Tu, MengChao Wu, HongYang Wang

**Affiliations:** 1 International Co-operation Laboratory on Signal Transduction, Eastern Hepatobiliary Surgery Institute, Second Military Medical University, Shanghai, China; 2 Department of Surgery, Eastern Hepatobiliary Surgery Hospital, Shanghai, China; 3 State Key Laboratory for Oncogenes and Related Genes, Cancer Institute of Ren Ji Hospital, Shanghai Jiao Tong University, Shanghai, China

## Abstract

**Background:**

Oxidative stress has been implicated in the pathogenesis of a wide spectrum of human diseases, including Hepatitis B virus (HBV)-related liver disease. Hepatitis B virus X protein (HBx) is a key regulator of HBV that exerts pleiotropic activity on cellular functions. Recent studies showed that HBx alters mitochondrial membrane potential, thereby sensitizing cells to pro-apoptotic signals. However, it remains largely unknown whether susceptibility of hepatocytes could be disturbed by HBx under oxidative stress conditions. The purpose of this study is to determine the apoptotic susceptibility of HBx-expressing hepatocytes upon exposure to pro-oxidant stimuli *in vitro *and *in vivo *and explore its underlying mechanism.

**Results:**

Although expression of HBx itself did not activate apoptotic signaling, it significantly enhanced oxidative stress-induced cell death both *in vitro *and *in vivo*. Interestingly, this phenomenon was associated with a pronounced reduction of protein levels of Mcl-1, but not other anti-apoptotic Bcl-2 members. Importantly, enforced expression of Mcl-1 prevented HBx-triggered cell apoptosis; conversely, specific knockdown of Mcl-1 exacerbated HBx-induced apoptosis upon exposure to oxidative stress. Furthermore, inhibition of caspase-3 not only abrogated HBx-triggered apoptotic killing but also blocked HBx-induced Mcl-1 loss. Additionally, expression of HBx and Mcl-1 was found to be inversely correlated in HBV-related hepatocellular carcinogenesis (HCC) tissues.

**Conclusions:**

Our findings indicate that HBx exerts pro-apoptotic effect upon exposure to oxidative stress probably through accelerating the loss of Mcl-1 protein via caspase-3 cascade, which may shed a new light on the molecular mechanism of HBV-related hepatocarcinogenesis.

## Background

Chronic Hepatitis B virus (HBV) infection is a major risk factor of human chronic liver disease and is strongly associated with hepatocellular carcinogenesis (HCC). Among the HBV encoding proteins, HBV X protein (HBx) is considered as a critical viral protein that exhibits multifunctional activities in modulating gene transcription, protein degradation, signal transduction, cell proliferation, cell cycle progress, senescence, autophagy and apoptosis [1-4].

Since apoptosis has been implicated as an important mechanism for liver injury [5,6], much effort has been made to understand the role of HBx in the regulation of apoptosis and its contribution to HCC. To date, the reported effects of HBx on apoptosis are controversial. As reported previously, the discrepancy of the role of HBx on cell apoptosis may be due to the different culture conditions and experimental systems used in these studies. Nevertheless, majority of these studies demonstrated that HBx can induce cell death or sensitize hepatocytes to a variety of apoptotic signals such as TNF-α, TRAIL, vitamin K3, ethanol, Fas, and UV [7-12]. In experimental animals, HBx transgenic mice also exhibit increased hepatic apoptosis [13].

It is well known that oxidative stress have been implicated in the pathogenesis of inflammatory diseases and cancer [14] and reactive oxygen species (ROS) are continuously generated within chronic inflammation and malignant tumor tissues. In addition, infiltration of activated phagocytic cells in liver disease provides another source of ROS production that promotes oxidative damage to hepatocytes [15]. Recent work showed that HBx expression could alter mitochondrial membrane potential and increase cellular ROS production, thereby sensitizing hepatoma cells to apoptotic stimuli [9,16]. Consistent with these *in vitro *findings, HBV transgenic mice also display elevated oxidative stress levels in the liver as compared to the nontransgenic control strain [17]. Thus, it is possible that, in HBV-infected liver, HBx protein and oxidative signals generated within the microenvironment may cooperate to increase cellular ROS accumulation up to a deleterious level, thereby resulting in overt liver cell damage. However, relatively little research has addressed the issue of whether susceptibility of hepatocytes upon exposure to oxidative stress could be affected by HBx.

The Bcl-2 protein family plays a pivotal role for mitochondrial membrane integrity and apoptosis regulation [18,19]. Among them, Mcl-1 is both structurally and functionally an anti-apoptotic member of the Bcl-2 family. It mainly locates on the outer membrane of mitochondria and is an important regulator of mitochondria-mediated apoptosis by preventing the release of cytochrome c into cytosol [20]. Recently, it has been demonstrated that Mcl-1 plays a key role in regulation of apoptosis and survival in multiple tissues and cell lines [21,22]. It is frequently overexpressed in several human malignancies such as multiple myeloma, non-small cell lung cancer and HCC. Knock down Mcl-1 protein expression sensitizes HCC cells towards apoptosis induction [23,24]. Using a conditional knock-out animal model, Schulze-Bergkamen H and his team demonstrated that hepatocyte-specific deletion of Mcl-1 not only increases spontaneous hepatocyte apoptosis resulting in profound liver cell damage and increases susceptibility of hepatocytes to pro-apoptotic stimuli [25], but also, more importantly, triggers hepatocellular proliferation and causes HCC [26]. Results from previous studies showed that H_2_O_2 _could abrogate the prosurvival function of Mcl-1 either by diminishing its levels or by inactivating its function [27,28], however, little is known about the potential role of Mcl-1 in HBx-induced cell killing. Given the importance of Mcl-1 in maintaining liver homeostasis, the aim of this work was to determine the apoptotic susceptibility of HBx-expressing hepatocytes under oxidative stress conditions and explore the possible role of Mcl-1 in this process.

Here, we reported that HBx enhanced oxidative stress-induced apoptotic killing both *in vitro *and *in vivo*, which is probably through accelerating the loss of Mcl-1 protein via caspase-3 cascade. Our results may have implications for understanding HBV-related hepatocarcinogenesis.

## Results

### HBx-Tg mice exhibit an increased oxidative stress and apoptotic susceptibility to liver ischemia-reperfusion challenge

**Figure 1:**
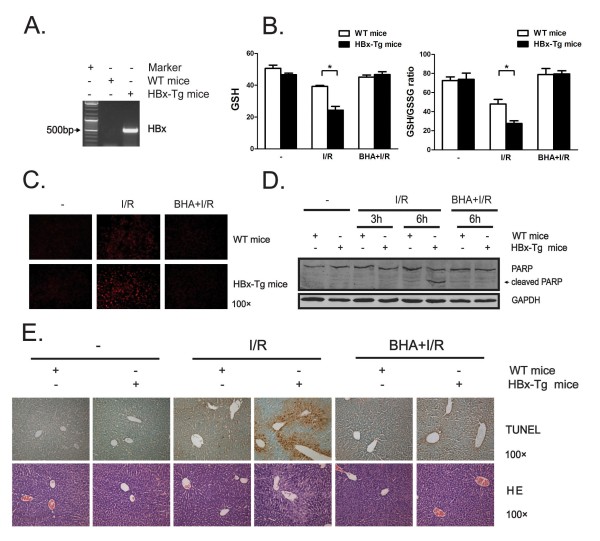
**HBx-Tg mice exhibit an increased oxidative stress and susceptibility to liver ischemia-reperfusion challenge**. (A) The transcript of HBx in livers from HBx-Tg or wide type (WT) mice was detected using RT-PCR. (B) Mice were given butylated hydroxyanisole (BHA)-containing (0.7%) or control diet for 2 days and then subjected to 60 min of warm liver ischemia (see "Materials and Methods"), followed by 6 hr of reperfusion before sacrificed. Total liver GSH content and GSH/GSSG ratio were determined. Values are mean ± SD (n = 4). *, p < 0.05. (C) Frozen liver sections from mice treated as in Fig. 1B were subjected to dihydroethidine (DHE) staining. Representative results are shown. Magnification, ×100. (D) Livers from mice treated as in Fig. 1B were homogenized and the protein levels of PARP and GAPDH were determined by Western blot assay. GAPDH was used as a loading control. (E) Liver sections from mice treated as in Fig. 1B were subjected to TUNEL and HE staining. Representative results are shown. Magnification, ×100.

To investigate whether susceptibility of hepatocytes under oxidative stress conditions could be disturbed by HBx *in vivo*, HBx transgenic (HBx-Tg) mice and wide type (WT) control strain were used (Figure 1A) and subjected to warm liver ischemia-reperfusion (I/R), an animal model which mimics pro-oxidant milieu *in vivo*. As expected, a decrease in total liver GSH level, an indicator of hepatocyte ROS accumulation, was observed in I/R-treated WT mice. Notably, an even greater decrease in liver GSH content was detected in I/R-treated HBx-Tg mice (Figure 1B). As an important index of oxidative stress, liver GSH/GSSG ratio were also monitored. Similarly, liver I/R treatment caused an even greater dramatic fall in the GSH/GSSG ratio in HBx-Tg mice than WT mice (Figure 1B). To evaluate in situ formation of ROS, the oxidative fluorescent dye dihydroethidine (DHE) was used by a method described by Sakurai T et al [29]. More extensive fluorescence was seen in livers of HBx-Tg mice than matched controls after liver I/R treatment (Figure 1C), indicating that HBx promotes cellular ROS accumulation upon oxidative stress stimulation. Meanwhile, increased hepatocyte apoptosis, as determined by PARP cleavage, was also observed in livers of HBx-Tg mice as compared to WT mice following I/R challenge (Figure 1D). To further evaluate hepatocyte apoptosis in the liver, a TUNEL-based immunohistochemistry assay was performed. Consistently, livers from HBx-Tg mice exhibited a pronounced accumulation of TUNEL-positive cells compared with those from WT mice following I/R treatment (Figure 1E). These findings confirm that HBx-Tg mice are more susceptible to oxidative stress-induced hepatocyte apoptosis.

To evaluate whether increased oxidative stress contribute to the pro-apoptotic effect of HBx, mice were given antioxidant butylated hydroxyanisole (BHA)-containing (0.7%) or control chow for 2 days and then challenged with liver I/R. Importantly, BHA administration not only restored the liver GSH content and GSH/GSSG ratio and reduced DHE-stained cells in I/R-treated HBx-Tg mice to a level similar to matched controls, but also effectively abrogated increased cell apoptosis in livers of I/R-challenged HBx-Tg mice (Figure 1B, C and 1D). Furthermore, histological analysis revealed that BHA treatment almost completely blocked appearance of TUNEL-positive hepatocytes in I/R-treated HBx-Tg mice (Figure 1E). Thus, HBx enhances oxidative stress-induced cell death through a mechanism likely to depend on ROS accumulation.

### HBx enhances cellular ROS production and sensitizes hepatocytes to H_2_O_2_-induced apoptosis

**Figure 2:**
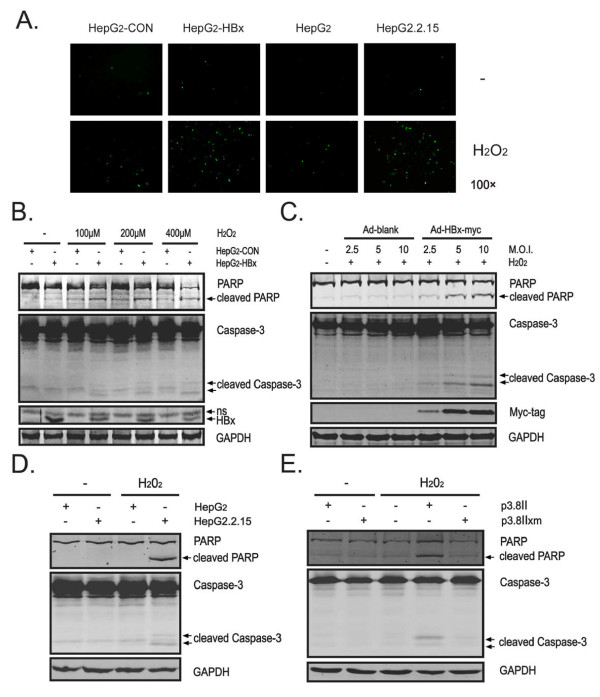
**HBx enhances production of cellular ROS and sensitizes hepatocytes to H_2_O_2_-induced apoptosis**. (A) Indicated cells were treated with or without H_2_O_2 _(400 μM) for 15 hr and then incubated with DCFH-DA for 30 min and the production of ROS was determined. Representative results are shown. Magnification, ×100. (B) Cells were exposed to the indicated amount of H_2_O_2 _for 15 hr and the protein levels of PARP, caspase-3, HBx and GAPDH were determined by Western blot assay. "ns", nonspecific bands. (C) HepG2 cells were infected with Ad-HBx-myc or Ad-blank at the indicated MOI for 36 hr followed by treated with H_2_O_2 _(400 μM) for additional 15 hr. Expression of PARP, caspase-3, Myc-tag and GAPDH was analyzed by Western blot assay. (D) HepG2.2.15 or HepG2 cells were treated with or without H_2_O_2 _(400 μM) for 15 hr and protein levels of PARP, caspase-3 and GAPDH were measured by Western blot assay. (E) SMMC-7721 cells were transiently transfected with p3.8II or p3.8IIxm for 36 hr followed by treated with or without H_2_O_2 _(400 μM) for additional 15 hr. Then cell lysates were subjected to Western blot and probed with indicated antibodies.

To further confirm the *in vivo *data, we employed HBx-expressing stable HepG2 (HepG2-HBx) cells and empty vector (HepG2-con) counterparts as described previously [30]. Consistent with our *in vivo *results, H_2_O_2 _exposure resulted in an increase in ROS levels in HepG2-con cells, but under the same condition, much more ROS-positive cells were seen in H_2_O_2_-exposed HepG2-HBx cells than control cells (Figure 2A). To examine whether the effect of HBx on ROS accumulation reflects the events in HBV-infected cells, we compared the ROS levels in parental HepG2 cells with HepG2.2.15 cells that constitutively replicated HBV upon exposure to H_2_O_2_. Similarly, HepG2.2.15 cells also exhibited a greater percentage of ROS-positive cells than parental HepG2 cells (Figure 2A).

To explore the role of ROS in the mechanism of HBx-sensitized cell apoptosis, cells were treated with H_2_O_2 _in concentrations from 100 to 400 μM. HBx-mediated cell death was found to increase after H_2_O_2 _exposure in a dose-dependent manner (Figure 2B). To evaluate the potential dose-effect relationship between HBx and apoptotic killing, a recombinant Myc-tagged HBx-expressing adenoviral system was used as described previously [30]. As expected, adenovirus-mediated gene transfer of HBx dose-dependently increased the susceptibility of HepG2 cells toward H_2_O_2_-induced apoptosis (Figure 2C).

Despite the evidence that apoptosis was apparent in the HBx-expressing cells, it is not sufficient to reflect what really happens during HBV infection as the level of HBx expression is usually low in HBV-infected cells and tissues. Therefore, we examined the apoptotic susceptibility of HepG2.2.15 cells upon oxidative stress stimulation. Consistently, H_2_O_2 _treatment induced significant apoptotic killing in HepG2.2.15 cells as compared to control cells (Figure 2D), supporting an apoptosis-promoting activity of HBV under oxidative stress conditions. To further determine whether HBx is required for HBV-induced cell death, SMMC-7721 cells were transfected with the p3.8II plasmid containing the wild-type HBV genome or with p3.8IIxm, an HBx-mutated HBV genome and then challenged with H_2_O_2 _stress. Strikingly, p3.8II-transfected cells showed an increased susceptibility to H_2_O_2_-induced apoptosis (Figure 2E), whereas p3.8IIxm-transfected cells showed significant apoptosis resistance in response to H_2_O_2 _stimulation, indicating that HBx is essential for HBV-induced apoptotic killing.

Together, these *in vitro *and *in vivo *data confirm that HBx enhances cellular ROS accumulation and triggers apoptosis under conditions of oxidative stress.

### HBx decreases the expression of the anti-apoptotic Mcl-1 protein upon oxidative stress stimulation

**Figure 3:**
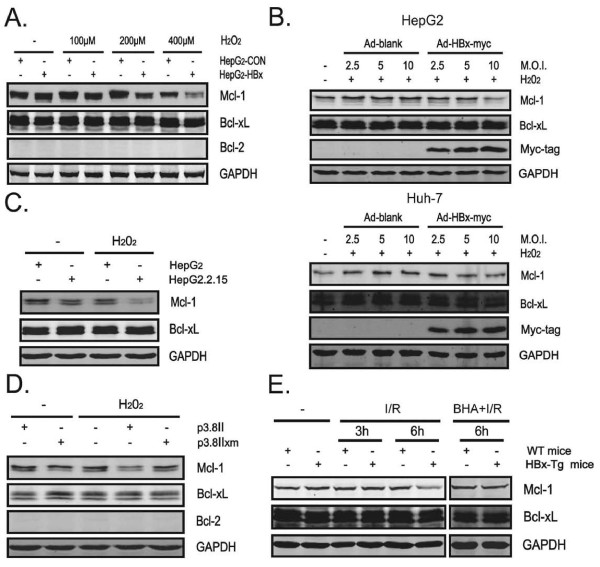
**HBx decreases the expression of the anti-apoptotic Mcl-1 protein upon oxidative stress stimulation**. (A) Cells were exposed to the indicated amount of H_2_O_2 _for 15 hr and expression of Mcl-1, Bcl-xL, Bcl-2 and GAPDH was analyzed by Western blot assay. (B) HepG2 or Huh-7 cells were infected with Ad-HBx-myc or Ad-blank at the indicated MOI for 36 hr followed by treated with H_2_O_2 _(400 μM) for additional 15 hr. Expression of Mcl-1, Bcl-xL, Myc-tag and GAPDH was analyzed by Western blot assay. (C) Cells were treated with or without H_2_O_2 _(400 μM) for 15 hr and indicated proteins were determined by Western blot assay. (D) SMMC-7721 cells were treated as in Fig. 2E and indicated proteins were determined by Western blot assay. (E) Livers from mice treated as in Fig. 1D were homogenized and protein levels of Mcl-1, Bcl-xL and GAPDH were determined by Western blot assay.

Next, we attempted to investigate the molecular events responsible for HBx-enhanced cell death upon exposure to oxidative stress. In view of the pivotal role that anti-apoptotic Bcl-2 family members play in mitochondrial integrity and hepatocyte survival [18,19], we examined expression of three important anti-apoptotic Bcl-2 family proteins (Bcl-2, Mcl-1, and Bcl-xL) in response to H_2_O_2_. Consistent with a previous report [31], Bcl-2 protein was not detected in hepatoma cell lines examined (Figure 3). Interestingly, protein levels of Mcl-1 declined significantly in HepG2-HBx cells compared with those in HepG2-con cells after H_2_O_2 _treatment in a dose-dependent manner, while Bcl-xL protein levels revealed no substantial difference between HepG2-HBx and HepG2-con cells (Figure 3A). In addition, ectopic expression of HBx significantly reduced Mcl-1 expression in HepG2 and Huh-7 cells, but had no major effect on Bcl-xL expression (Figure 3B). Furthermore, Mcl-1 expression decreased greatly in HBV-replicating HepG2.2.15 cells compared with that in parental HepG2 cells after treatment with H_2_O_2 _(Figure 3C), suggesting that HBV may have a similar effect on Mcl-1 expression as HBx does. Importantly, protein levels of Mcl-1 significantly reduced in p3.8II-transfected but not p3.8IIxm-transfected SMMC-7721 cells upon H_2_O_2 _exposure, suggesting that HBx is also essential for HBV to promote the loss of Mcl-1 protein (Figure 3D). To further confirm these *in vitro *findings, mice were challenged with liver I/R treatment and Mcl-1 expression in livers of HBx-Tg and WT mice was determined by Western blot assay. As illustrated in Figure 3E, levels of Mcl-1 were also found to decrease in livers of I/R-challenged HBx-Tg mice as compared to matched controls. Additionally, antioxidant BHA pretreatment abrogated the loss of Mcl-1 protein in livers of I/R-treated HBx-Tg mice, suggesting that HBx-mediated diminished expression of Mcl-1 under oxidative stress conditions is mainly ROS dependent. Collectively, HBx accelerates the loss of Mcl-1 protein in response to oxidative stress both *in vitro *and *in vivo*.

### Reduction of Mcl-1 is involved in pro-apoptotic effect of HBx in response to oxidative stress

**Figure 4:**
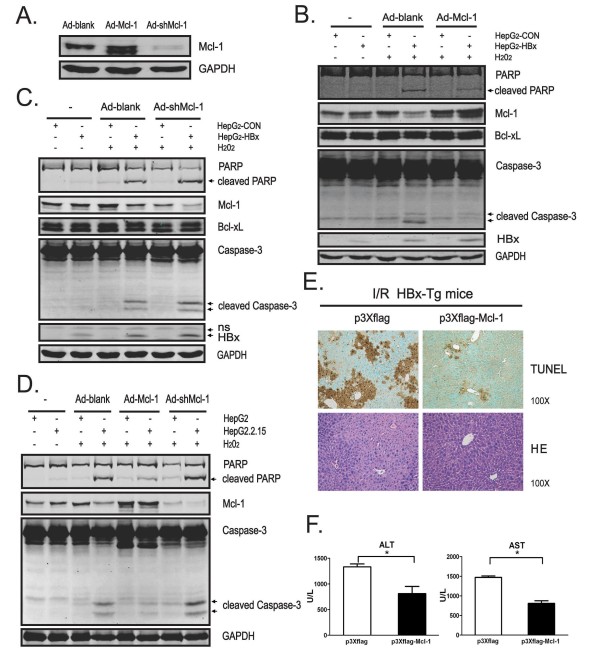
**Reduction of Mcl-1 is involved in pro-apoptotic effect of HBx in response to oxidative stress**. (A) HepG2 cells were infected with Ad-Mcl-1, Ad-shMcl-1 or Ad-blank (M.O.I.= 5) for 36 hr and protein levels of Mcl-1 and GAPDH were determined by Western blot assay. (B, C and D) Cells were infected with indicated adenoviruses (M.O.I. = 5) for 36 hr followed by exposed to H_2_O_2 _(400 μM) for additional 15 hr. Expression of PARP, caspase-3, Mcl-1, Bcl-xL, HBx and GAPDH was evaluated by Western Blot assay. "ns", nonspecific bands. (E) HBx-Tg mice were subjected to a single injection of Mcl-1-expressing plasmid (p3×flag-Mcl-1) or control plasmid (p3×flag) by tail vein using a hydrodynamics-based procedure (see "Materials and Methods"). Three days later, mice were challenged with liver I/R. Livers were collected and subjected to TUNEL and H.E. staining. Representative results are shown. Magnification, ×100. (F) Mice were treated as in Fig. 4E and circulating levels of serum ALT and AST were analyzed. Results are mean ± SD (n = 4). *, p < 0.05.

**Figure 5:**
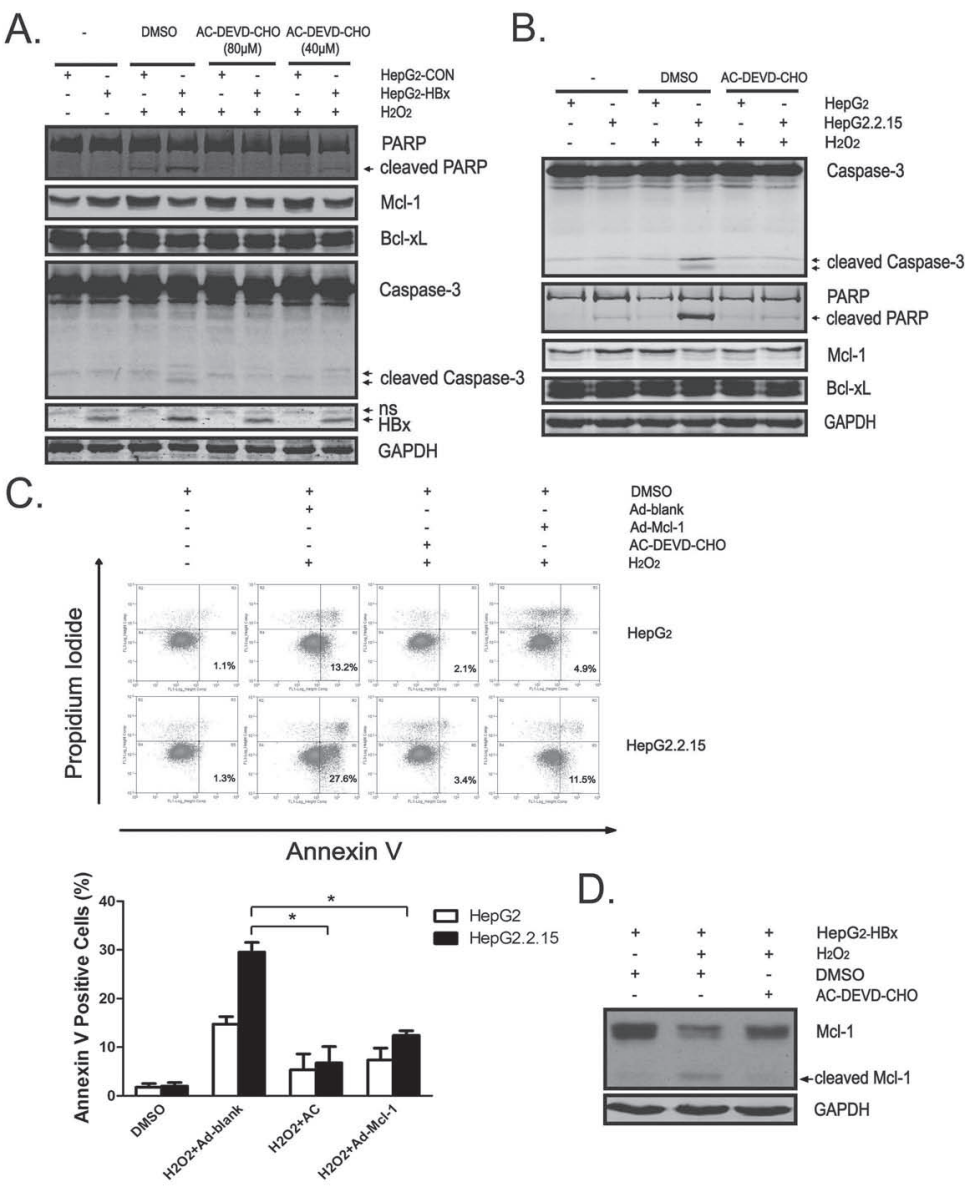
**The caspase-3 inhibitor prevents loss of Mcl-1 in HBx-expressing cells upon H_2_O_2 _exposure**. (A and B) Cells were treated with or without indicated amount of AC-DEVD-CHO for 5 hr followed by exposed to H_2_O_2 _(400 μM) for additional 15 hr. Expression of PARP, caspase-3, Mcl-1, Bcl-xL, HBx and GAPDH was determined by Western Blot assay. "ns", nonspecific bands. (C) Cells were infected with indicated adenoviruses (M.O.I. = 5) for 36 hr or treated with DMSO or AC-DEVD-CHO (80 μM) for 5 hr followed by exposed to H_2_O_2 _(400 μM) for additional 15 hr. The extent of apoptosis was evaluated by FACS analysis. Representative results are shown. Plots (lower panel) are mean ± SD of data from three independent experiments. *, p < 0.05. "AC", AC-DEVD-CHO. (D) Cells were treated with DMSO or AC-DEVD-CHO (80 μM) for 5 hr followed by exposed to H_2_O_2 _(400 μM) for additional 12 hr. Expression of Mcl-1 was determined by Western Blot assay. A band at about 28 kDa indicates the cleaved form of Mcl-1.

To determine whether loss of Mcl-1 plays a role in HBx-mediated apoptotic killing under oxidative stress conditions, Mcl-1-expressing adenovirus (Ad-Mcl-1) and Mcl-1-shRNA adenovirus (Ad-shMcl-1), which specific knockdown of Mcl-1 expression, were developed (Figure 4A). Importantly, enforced expression of Mcl-1 profoundly attenuated caspase-3 activation and PARP cleavage in H_2_O_2_-treated HepG2-HBx cells compared with control cells (Figure 4B). Conversely, Adenovirus-mediated siRNA targeting Mcl-1 gene further exacerbated the activation of caspase-3 and cleavage of PARP in HepG2-HBx cells upon H_2_O_2 _exposure (Figure 4C). Consistently, similar results were also obtained in HepG2.2.15 cells in which over-expression of Mcl-1 prevented the apoptotic cell death in H_2_O_2_-treated HepG2.2.15 cells, while knockdown of Mcl-1 further increased the apoptotic susceptibility of HepG2.2.15 cells toward H_2_O_2 _stress (Figure 4D). Quantification of annexin-V-stained cells by FACS analysis further corroborated this finding: increased apoptosis in H_2_O_2_-treated HepG2.2.15 cells was significantly attenuated by Mcl-1 over-expression (Figure 5C).

To further evaluate the role of Mcl-1 down-regulation in HBx-mediated cell death under oxidative stress conditions *in vivo*, HBx-Tg mice were administered Mcl-1-expressing plasmid (p3×flag-Mcl-1) or control plasmid (p3×flag) by tail vein injection, and Mcl-1 expression was confirmed in livers from p3×flag-Mcl-1-treated mice (Additional file 1 Figure S1). Three days later, mice were subjected to warm liver I/R challenge. As expected, TUNEL assay and serum ALT and AST examination showed that I/R challenge-induced liver injury in HBx-Tg mice was greatly improved by Mcl-1-expressing plasmid administration (Figure 4E and 4F). Thus, hepatocytes from HBx-Tg mice are more susceptible to oxidative stress-induced apoptosis, at least in part, through accelerating the loss of Mcl-1 protein. These findings support the notion that reduction of Mcl-1 is required for pro-apoptotic effect of HBx under oxidative stress conditions.

### The caspase-3 inhibitor prevents loss of Mcl-1 in HBx-expressing cells upon H_2_O_2 _exposure

It has been reported that caspase-3-mediated proteolysis may contribute to diminished expression of Mcl-1 in some cell types [32-35]. We next investigated the effects of caspase-3 inhibitor for its ability to modulate HBx-enhanced Mcl-1 loss. Strikingly, caspase-3-specific inhibitor AC-DEVD-CHO not only prevented the activation of caspase-3 and cleavage of PARP, but also attenuated the loss of Mcl-1 protein in H_2_O_2_-exposed HepG2-HBx cells in a dose-dependent manner (Figure 5A). Similarly, incubation of cell with AC-DEVD-CHO not only protected HepG2.2.15 cells against H_2_O_2_-induced apoptosis, but also inhibited the observed reduction in Mcl-1 expression in H_2_O_2_-treated HepG2.2.15 cells (Figure 5B and 5C). The above experiments indicated that HBx may trigger caspase-3-mediated Mcl-1 turnover during H_2_O_2 _treatment because of the ability of the caspase-3 inhibitor to prevent turnover. It was therefore important to investigate whether cleaved products of Mcl-1 could be detected in HBx-expressing cells following H_2_O_2 _treatment and whether cleavage of Mcl-1 could be prevented by caspase-3 inhibitor. Following treatment with H_2_O_2 _for 12 hr, a band at approximately 28 kDa was detected in HepG2-HBx cells using anti-Mcl-1 antibody, and this may be attributed to caspase-cleaved product of Mcl-1. Importantly, caspase-3 inhibitor AC-DEVD-CHO prevented the appearance of this band and restored protein levels of full-length Mcl-1 in H_2_O_2_-treated HepG2-HBx cells, suggesting that HBx triggers loss of Mcl-1 protein mainly through caspase-3-mediated cleavage (Figure 5D). Of note, levels of this cleavage product decreased thereafter (data not shown), in agreement with some previous reports in some systems [34], indicating that the caspase-cleaved product of Mcl-1 in HepG2-HBx cells in this scenario may be not stable. Thus, loss of Mcl-1 in HBx-expressing cells exposed to oxidative stress is mainly caspase-3 dependent.

### Expression of HBx and Mcl-1 is inversely correlated in HBV-related HCC tissues

**Figure 6:**
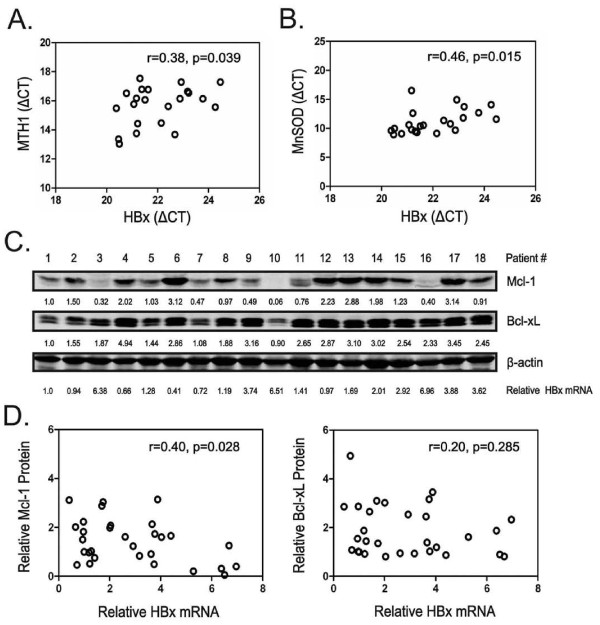
**Expression of HBx and Mcl-1 is inversely correlated in HBV-related HCC tissues**. (A) The correlation of HBx and hMTH1 mRNA expression and (B) HBx and MnSOD mRNA expression in 22 HBV-positive HCC samples was shown. (C) Protein levels of Mcl-1, Bcl-xL and β-actin were determined by Western blot assay in 18 HBV-positive HCC samples. The relative protein expression of Mcl-1 and Bcl-xL was quantified and normalized to β-actin. The relative mRNA expression of HBx was also quantified and normalized to 18S rRNA. (D) The relationship between HBx and Mcl-1 expression or HBx and Bcl-xL expression in 30 HBV-positive HCC samples was shown. Correlation between two variables was calculated by Spearman rank correlation coefficient (r).

Malignant tumors, including HCC, are frequently under persistent oxidative stress, and alterations in DNA repair enzymes and antioxidant enzymes are considered as indicators of oxidative damage [36,37]. To evaluate the potential relationship between HBx and oxidative stress in HBV-related HCC tissues, expression of HBx, DNA repair enzyme human MutT homolog 1 (hMTH1) and antioxidant enzyme manganese superoxide dismutase (MnSOD) was analyzed in HBV-positive HCC tissues. As illustrated in Figure 6A, a positive correlation between HBx and hMTH1 mRNA expression was observed in 22 HCC samples (r = 0.38, p = 0.039). In addition, HBx mRNA expression was also significantly correlated to MnSOD mRNA expression in these HCC samples (r = 0.46, p = 0.015) (Figure 6B). These results suggest that there may be a correlation between HBx expression and the extent of oxidative damage in HBV-positive HCC tissues.

To further examine the relationship between HBx and anti-apoptotic Bcl-2 family members in HBV-related HCCs, protein levels of Mcl-1 and Bcl-xL in HBV-positive HCC tissues were evaluated by Western blot analysis (Figure 6C). Since HBx protein was hardly detectable in HCC tissues, HBx expression was determined by its mRNA levels. As expected, HBx mRNA expression was found to be inversely correlated with Mcl-1 protein expression in 30 HCC samples (r = 0.40, p = 0.028) However, no correlation was observed between HBx and Bcl-xL expression (r = 0.20, p = 0.285) (Figure 6D). Taken together, these histological observations, along with the above findings, suggest a potential pathophysiological role of Mcl-1 in HBV-associated hepatocarcinogenesis.

## Discussion

Persistent oxidative stress has been suggested to be a major contributor to the development of HCC as it can exert multiple pro-tumorigenic effects, including altered gene expression [38], epigenetic modulations [37], enhanced hepatocyte death and IL-1α release [29], genomic instability [39] as well as higher DNA mutation rates [40]. Given the critical role of HBx in the pathogenesis of HBV-related liver cancer [1,2] and Mcl-1 in liver homeostasis [25,26,41], the purpose of this study was to determine whether the apoptotic susceptibility of hepatocytes under oxidative stress conditions could be disturbed by HBx and the potential role of Mcl-1 in this process.

In the present study, the concentration of H_2_O_2 _we used did not induce apparent apoptosis in control hepatocytes, while it did trigger significant apoptotic killing in HBx-expressing hepatocytes. Consistently, no major injury was detected in WT control mice following liver I/R challenge, while severe liver cell death were observed in HBx-Tg mice receiving the same treatment. Furthermore, HBV-replicating HepG2.2.15 cells and SMMC-7721 cells transfected with the wild-type, but not HBx-mutated, HBV genome unanimously exhibited increased apoptotic susceptibility to H_2_O_2 _stress. These *in vitro *and *in vivo *data clearly demonstrated that HBx expression sensitized hepatocytes to oxidative stress-mediated cell killing. Our findings agree with the majority results of previous observations which showed that HBx increases susceptibility of hepatocytes to a variety of apoptotic stimuli [7-12], although there are opposite data about the effects of HBx protein on apoptotic signals [2,42]. The discrepant activity of HBx on apoptosis may result from the different stages of natural HBV infection. It is possible, as proposed by Arbuthnot P and his colleagues [43], that HBx inhibits apoptosis at early stage during hepatocyte infection in order to facilitate HBV replication, while promotes apoptosis at later stage to accelerate virus spread.

Recent work showed that HBx mainly localizes in the mitochondria, disturbs mitochondrial membrane potential and subsequently increases ROS production, suggesting that HBx has an ability to activate mitochondria-dependent apoptosis. Given the critical role that anti-apoptotic Bcl-2 family proteins play in liver homeostasis and apoptosis control [18,19], we went further to examine the expression of anti-apoptotic Bcl-2 members. Bcl-xL and Mcl-1 have been identified as major anti-apoptotic Bcl-2 proteins in the liver for previous studies revealed that Bcl-2 is not generally expressed in human hepatocytes and hepatoma cell lines [31]. Both of these proteins play a crucial role for the maintenance of mitochondrial membrane integrity. They mainly localize at the outer membrane of mitochondria, prevent the oligomerization and the activation of multidomain pro-apoptotic proteins Bax and Bak [44,45]. In addition, they can also inhibit apoptosis by sequestering pro-apoptotic BH3-only proteins such as Bid, Bim or Puma [46]. Interestingly, however, only protein levels of Mcl-1 were found to decrease significantly in HBx-expressing cell lines and livers of HBx-Tg mice upon oxidative stress stimulation, while no major changes in Bcl-xL levels were observed. Consistently, analysis from clinical samples also revealed an inverse correlation between the expression of HBx and Mcl-1, but not Bcl-xL, in HBV-related HCC tissues. Thus, these results argue against the involvement of Bcl-xL in HBx-mediated cell killing in response to oxidative stress and suggest that different anti-apoptotic Bcl-2 members may not functionally equivalent under certain conditions [46,47]. Furthermore, similar results were also obtained in HepG2.2.15 cells as well as p3.8II-transfected but not p3.8IIxm-transfected SMMC-7721 cells. Consistent with a previous report [48], we also observed a mild increase in Mcl-1 protein levels in unstimulated HepG2-HBx cells compared with those in HepG2-con cells, and this may be due to the transactivation effects of HBx. Nevertheless, upon H_2_O_2 _exposure, levels of Mcl-1 declined significantly in HepG2-HBx cells as compared to matched controls, indicating that rapid loss of this highly regulated protein may be involved in HBx-mediated cell killing. Indeed, enforced expression of Mcl-1 provided long-term protection against HBx-induced apoptosis. Conversely, specific knockdown of Mcl-1 expression further exacerbated HBx-induced apoptosis. It should be noted that the protection effect of Mcl-1 over-expression was relatively weaker than that of caspase-3 inhibition, indicating that down-regulation of Mcl-1 may not be the exclusive pathway that mediates the pro-apoptotic activity of HBx.

Ahmad KA and colleague have demonstrated that Bax plays an important role in H_2_O_2_-induced apoptosis via mitochondrial translocation [49], meanwhile Mcl-1 has been shown to interact with Bak and prevent its translocation to the mitochondria [44,45]. We therefore examined the expression and mitochondrial translocation of Bax/Bak in our system. Although H_2_O_2 _did trigger the translocation of Bax/Bak from the cytosol to the mitochondria, a reduced expression of Bax/Bak was observed in both the whole cell lysates and their mitochondria fractions of HBx-expressing cells as compared to matched control cells (Additional file 1 Figure S2), which appears to be inconsistent with the previous report [49]. Nevertheless, we speculated that, after 15 hr of treatment with H_2_O_2_, HBx-expressing cells may undergo relatively late-stage apoptosis, which may lead to protease hydrolysis of Bax/Bak or trigger some potential mechanisms to dysregulate their expression. These observations are in agreement with an earlier study [50]. Moreover, the cleavage of Bax was also detected under this condition (data not shown). As we did not examine the expression and mitochondrial translocation of Bax/Bak at earlier intervals, it is unlikely to rule out the possible involvement of Bax or Bak in HBx-enhanced cell death, yet, our findings strongly support the notion that Mcl-1 plays a functional role in HBx-mediated apoptotic killing under oxidative stress conditions.

Expression of Mcl-1 is tightly controlled through diverse signaling pathways. Results of the present study identified the association of Mcl-1 down-regulation with caspase activation, as caspase-3 inhibitor AC-DEVD-CHO not only blocked HBx-mediated apoptosis but also significantly attenuated the observed reduction of Mcl-1 expression in ROS-exposed HBx-expressing cells. These findings are consistent with our previous study, which demonstrated that HBx protein renders hepatocytes susceptible to chemotherapeutic agent cisplatin through stimulating oxidative stress-dependent caspase-3-mediated degradation of Mcl-1 [30]. Moreover, in this study, we further showed that HBx actually has the ability to sensitize hepatocytes to oxidative signals *per se*-induced apoptosis, and that this pro-apoptotic effect of HBx was also mediated through accelerating caspase-3-dependent loss of Mcl-1 protein. Furthermore, we also detected a caspase-cleaved product of Mcl-1 in H_2_O_2_-treated HepG2-HBx cells, as this cleavage of Mcl-1 could be prevented by caspase-3 inhibitor. Given these observations, we propose that caspase-3-mediated degradation of Mcl-1 may represent a common mechanism during pro-oxidant stimuli-induced apoptosis in HBx-expressing cells.

Of note, although caspase-3 inhibitor greatly prevented Mcl-1 loss in H_2_O_2_-treated HepG2-HBx cells, it did not completely restore its protein levels as compared to unstimulated HepG2-HBx cells (Figure 5D), indicating that other mechanism may also contribute to reduce Mcl-1 expression. Inoshita S and coworkers [28] reported that short-term exposure (1~3 hr) of HEK293 and PAE cells to hydrogen peroxide results in JNK activation, which leads to apoptosis through phosphorylation and inactivation of Mcl-1, while they did not explore the effect of long-term H_2_O_2 _exposure on Mcl-1 expression. In the present study, we noticed that over 12 hr exposure of HBx-expressing hepatocytes to H_2_O_2 _caused a significant decrease in cellular Mcl-1 levels, and we also observed sustained activation of JNK in this setting (data not shown). As the ability of HBx to activate JNK pathway has been reported by several groups [51,52], future study should be warranted to determine the possible involvement of JNK signaling in HBx-triggered loss of Mcl-1 protein.

The observation that caspase-3 inhibitor prevented the loss of Mcl-1 protein in H_2_O_2_-exposed HBx-expressing cells indicates that although Mcl-1 mainly functions upstream of caspases, the major regulation of Mcl-1 by HBx following H_2_O_2 _treatment lies downstream of caspase-3. Thus, the reduction of full-length Mcl-1 protein levels due to caspase-3-mediated proteolysis represents a secondary rather than a primary event in the induction of cell death. We suppose that, under oxidative stress conditions, HBx may activate caspase-3 signaling through a Mcl-1-independent mechanism, and activated caspase-3 triggers down-regulation of full-length Mcl-1 protein through proteolysis, thus resulting in the impairment of the inhibitory effect of this anti-apoptotic molecule on mitochondria-dependent apoptosis and subsequent caspase-3 activation. As a result, caspase-3 cascade is further activated in a positive feedback loop, allowing the irreversible commitment to cell death. Recently, caspase-mediated proteolysis of Mcl-1 has been confirmed by several independent groups [32-35], however, it remains unclear whether the caspase-3 cleavage sites in Mcl-1 protein in HBx-expressing hepatocytes are still the same (Asp127 and Asp157) as reported previously [33], future studies based on site-directed point mutations and sequence analysis would help to address this issue. Moreover, it will be important to elucidate which signaling pathway is responsible for HBx-mediated caspase-3 activation under oxidative stress conditions.

In line with several previous reports [9,53], a slightly increased level of oxidative stress was observed both in unstimulated HepG2-HBx and HepG2.2.15 cells as well as livers of HBx-Tg mice as compared to their respective controls. Strikingly, upon moderate oxidative stress stimulation, HBx greatly enhanced the extent of oxidative stress, which was accompanied by a significant increase in the rate of apoptosis in HBx-expressing cells both *in vitro *and *in vivo*. Thus, HBx protein and oxidative signals may synergize to augment cellular ROS accumulation up to a deleterious level resulting in apparent liver injury. Antioxidant treatment effectively abrogated the loss of Mcl-1 protein and cell death in HBx-expressing cells, suggesting that pro-apoptotic effect of HBx under oxidative stress conditions is mainly ROS dependent. The fact that HBx-mediated cell killing was not apparent until moderate pro-oxidant stimuli were administrated indicates that a certain threshold of ROS level would be required to trigger apoptosis and that HBx itself may not be sufficient to initiate apoptotic event, but rather function as an apoptosis inducer under stress conditions [9,47]. Thus, results of the present work highlighted the importance of ROS accumulation in the pro-apoptotic activity of HBx, nevertheless, the exact mechanism by which HBx significantly stimulates cellular ROS production remains to be elucidated.

Controlled hepatocyte apoptosis is essential for liver homeostasis. However, uncontrolled apoptosis can induce compensatory proliferation of hepatocytes [25,26]. Although the ideas that increased hepatocyte apoptosis can cause liver cancer are seemingly inconsistent with the known phenomenon of apoptosis resistance of pre-malignant and malignant hepatocytes, uncontrolled hepatocyte apoptosis can lead to compensatory hepatocyte proliferation and cause HCC in animal model [26]. It is known that chronic viral hepatitis is characterized not only by inflammation, but also by an increased rate of apoptosis and elevated caspase activities to preserve homeostasis after tissue damage [54]. Therefore, it is likely that, as has been suggested by others [7], enhanced hepatocyte death triggered by HBx may promote the induction of liver cell growth factors, which in turn enhance compensatory hepatocyte proliferation, not only creating a larger reservoir of new uninfected hepatocytes to propagate the viral infection but also contributing to the development of HCC.

Mcl-1 was found to be highly expressed in human HCC, and has been implicated in the apoptosis resistance of HCC cells [23,24]. Thus, Mcl-1 seemingly plays a contradictory role in hepatocarcinogenesis. However, the role that Mcl-1 plays in HCC promotion and progression may depend on the milieu. It has long been known that chronic inflammation and tumor tissues are frequently under oxidative stress [14,55,56]. Hence, it is possible that, on one side, acceleration of Mcl-1 loss in HBV-infected hepatocytes under moderate or sublethal oxidative stress conditions may result in a pro-apoptotic environment provoking compensatory proliferation, finally giving rise to the outgrowth of the neoplastic cell population and contributing to the initiation of liver cancer [26]. On the other side, malignant hepatocytes that over expresses Mcl-1 can be selected during tumor progression and eventually confer resistance of HCC cells to apoptosis triggers. Our findings are also in agreement with a tumor promoting effect of a pro-oxidant intracellular milieu. For example, Clément MV and his groups demonstrated that overexpression of Bcl-2 increases intracellular O_2_^- ^and inhibits apoptotic acidification and cell death, while decrease in intracellular superoxide sensitizes Bcl-2-overexpressing tumor cells to apoptotic killing [57]. Consistently, Pervaiz, S and coworkers reported that GTP-binding protein Rac induces production of superoxide, thereby inhibiting tumor cell response to apoptosis, conversely, inhibition of the Rac pathway causes a decrease in superoxide anion concentration, resulting in a significant increase in tumor cell sensitivity to apoptosis [58].

## Conclusions

In conclusion, we provide both *in vitro *and *in vivo *evidence that HBx has the ability to enhance the susceptibility of hepatocytes toward oxidative stress-induced apoptotic killing by accelerating the loss of Mcl-1 protein, which is mainly caspase-3 dependent. Therefore, tissue microenvironments generating ROS such as chronic inflammation and injury may aggravate the pathogenesis of HBV-related liver disease by provoking cell death.

## Materials and methods

### Antibodies and Reagents

The primary antibodies specific for Mcl-1 (S-19 and K-20), Bax (N-20) and Bak (N-20) were purchased from Santa Cruz Biotechnology. Antibodies for Bcl-xL, Bcl-2, Caspase-3, PARP, GAPDH and Myc-tag were from Cell Signaling Technology (CST). Purified anti-Mcl-1 antibody was from Biolegend. Rabbit polyclonal anti-HBx antibody was generated in our laboratory. peroxide hydrogen (H_2_O_2_), butylated hydroxyanisole (BHA), 4',6'-Diamidino-2-phenylindole (DAPI) were from Sigma-Aldrich; AC-DEVD-CHO were from Calbiochem.

### Cell Lines and Cell Culture

HepG2, Huh-7 and HEK293A cell lines were obtained from American Type Culture Collection (Manassas, VA). SMMC-7721 and HepG2.2.15 cell lines were from the Cell Research Institute of Chinese Academy of Sciences (Shanghai, China). Cells were maintained at 37°C in a humidified incubator containing 5% CO_2 _in Dulbecco's modified Eagle's medium supplemented with 10% heat-inactivated fetal bovine serum and passed every 2-3 d to maintain logarithmic growth.

### Plasmids and Recombinant Adenovirus Preparation

The Myc-tagged full-length HBx plasmid (pcDNA3.1-HBx-myc) was constructed by inserting a PCR-amplified full-length HBx fragment into the EcoRI/KpnI sites of pcDNATM3.1/myc-His(-)A (Invitrogen), using the primers, forward, 5'-CGGAATT-CATGGCTGCTAGGCTGTGCTG-3' and reverse, 5'-GGGGTACCGGCAGA-GGTGAAAAAGTTGC-3'. The Mcl-1-expressing plasmid (p3×flag-Mcl-1) was generously provided by Prof. Wu Mian (University of Science and Technology of China, Hefei, China). The control (Ad-blank), Myc-tagged HBx-expressing (Ad-HBx-myc), Mcl-1-expressing (Ad-Mcl-1) and Ad-shMcl-1 (harboring Mcl-1-small hairpin RNA, Mcl-1-shRNA) recombinant adenoviruses were generated as described previously [30]. The primers were as follows: pAd-HBx-myc, forward, 5'-CGG-AATTCATGGCTGCTAGGCTGTGCTG-3'; reverse, 5'-GAAGATCTAAGCT-GGAGACCGTTTAAAC-3'; pAd-Mcl-1, forward, 5'-GGAATTCATGTTTGGC-CTCAAAAGAAACGCGG-3'; reverse, 5'-CGGGATCCGTCAACTATTGCACTT-ACAGTAAGG-3'. Mcl-1-shRNA was generated using the pSUPER RNAi System™ (Promega). The Mcl-1 siRNA sequence used was included in the following sense and antisense oligonucleotides: 5'-GATCCCCCGGGACTGGCTAGTTAAACTTC-AAGAGAGTTTAACTAGCCAGTCCCGTTTTTA-3' and 5'-AGCTTAAAAAC-GGGACTGGCTAGTTAAACTCTCTTGAAGTTTAACTAGCCAGTCCCGGGG-3'. Sense and antisense strands were annealed and ligated into the linearized pSUPER.neo+GFP Vector following the manufacturer's directions. All the constructs were confirmed by DNA sequencing and Western blot analysis. The recombinant adenovirus was generated in HEK293A cells by homologous recombination system. Adenovirus was purified using Adeno-X™ Virus Purification Kit (Clontech Laboratories). The titer of the virus was determined using Adeno-X™ Rapid Titer Kit (Clontech Laboratories) following the manufacturer's instructions.

### Transient transfection

SMMC-7721 cells were transiently transfected using PEI (Polyplus; AFAQ) as described previously [59].The plasmid p3.8II containing the wild-type HBV genome and p3.8IIXm consisting of an HBx-mutated HBV genome were kindly provided by Prof. Zhao Mujun (Institute of Biochemistry and Cell Biology, Shanghai Institutes for Biological Sciences, Chinese Academy of Sciences, Shanghai, China) [3].

### Semi-Quantitative and Real-Time Reverse Transcription-PCR

Total RNAs were isolated from cells or HCC samples using TRIzol Reagent (Invitrogen) following the manufacturer's instructions. The complementary DNA template was prepared using random primers and Moloney Murine Leukemia Virus reverse transcriptase (Promega) according to the manufacturer's protocol. After the reverse transcription reaction, the complementary DNA template was either semi-quantitated by reverse transcription-PCR (RT-PCR) or quantitated using real-time PCR technology. The primers used in this study are as follows: HBx (AY310322) forward, 5'-ATGGCTGCTAGGCTGTGCTG-3'; reverse, 5'-GGCAGAGGTGAA-AAAGTTGC-3'; hMTH1 (AK026631) forward, 5'-AGCCTCAGCGAGTTCT-CCTG-3'; reverse, 5'-GATCTGGCCCACCTTGTGC-3'; hMnSOD (Y00472) forward, 5'-GAGATGTTACAGCCCAGATAGC-3'; reverse, 5'-AATCCCCAGCAGTGGAA-TAAGG-3'; 18sRNA (NR_003286) forward, 5'-CGGCTACCACATCCAAGGAA-3'; reverse, 5'-GCTGGAATTACCGCGGCT-3'. 18srRNA was used as a control. Each sample was tested in duplicate.

### Western Blot Analysis

Western blotting was performed as described previously [60]. Briefly, whole-cell extracts or tumor specimens were prepared in lysis buffer [Tris-HCl (20 mM), pH 7.4, NaCl (150 mM), glycerol (10%), Nonidet P-40 (0.2%), EDTA (1 mM), EGTA (1 mM), PMSF (1 mM), NaF (10 mM), aprotinin (5 mg/ml), leupeptin (20 mM), and sodium orthovanadate (1 mM)] and centrifuged at 12,000 g for 15 min. Protein concentrations were measured using the BCA assay. Immunoblotting was performed using specific primary antibodies and immunocomplexes were incubated with the appropriate horseradish-peroxidase-conjugated secondary antibodies or fluorescein-conjugated secondary antibody, and then detected using the ECL kit (Santa Cruz Biotech) or Odyssey fluorescence scanner (Li-Cor, Lincoln, NE).

### Reactive Oxygen Species and Glutathione Measurement

Production of ROS was measured with the fluorogenic dye 2', 7'-dichloro-fluorescin diacetate (DCFH-DA), a cell permeant compound, using Reactive Oxygen Species Assay Kit (Invitrogen). Briefly, Cells were preincubated with DCFH-DA (10 μM) for 30 min at 37°C. After the extracellular dye was removed, the cells were washed 3 times and incubated with serum-free DMEM. Subsequently, fluorescence was measured at 488 nm excitation and 525 nm emission using a fluorescence microscope (Olympus). Total liver glutathione (GSH) content were determined by a commercial kit (Jiancheng, Nanjing, China) according to the manufacturer's protocol. GSH and GSSG Levels were measured using a GSH and GSSG Assay Kit (Beyotime, China). Liver in situ ROS production were determined by staining frozen liver sections with dihydroethidine (DHE) (Invitrogen), whose oxidation leads to the fluorescent derivative ethidine [29].

### Apoptosis Analysis

For apoptosis analysis, cells were seeded into 6-well plates with 5 × 10^5 ^cells/well and incubated overnight followed by treatment with or without H_2_O_2_. The extent of apoptosis was determined by FACS analysis (Beckman Coulter) using Annexin V Apoptosis Detection Kit (Invitrogen). Apoptotic cells in the liver were detected by terminal deoxynucleotidyl transferase dUTP nick end labeling (TUNEL) staining using In Situ Apoptosis Detection Kit (Calbiochem), and the nucleus was counterstained with methyl green.

### Preparation of cytosolic and mitochondria fractions

Preparation of cytosolic and mitochondria fractions was achieved using a commercially available cytosol/mitochondria fractionation kit according to the manufacturer's protocol (Beyotime, China). Briefly, 1 × 10^7 ^cells were washed with ice-chilled PBS at 1,200 g. Cell pellets were resuspended in 500 μL of extraction buffer and incubated at 4°C for 20 minutes, followed by homogenization. The homogenate was centrifuged at 1,000 g for 10 minutes at 4°C. The supernatant was additionally centrifuged at 3,500 g for 10 minutes (fraction enriched with intact mitochondria). The supernatant from the last centrifugation was used as the cytosolic fraction and the final pellet represents a more purified mitochondrial fraction.

### Liver Ischemia

HBx transgenic mice were kindly provided by Prof. Yang Xiao (Genetic Laboratory of Development and Diseases, Institute of Biotechnology, Beijing, China). The identification of HBx transgenic mice was performed as described previously [61]. A nonlethal model of segmental (70%) hepatic warm ischemia was used. All structures in the portal triad (hepatic artery, portal vein, bile duct) to the left and median liver lobes were occluded with a microvascular clamp for 60 min; reperfusion was initiated by removal of the clamp. At the end of the observation period, mice were sacrificed by cervical dislocation.

### In Vivo Gene Expression Experiments

Plasmid DNA was administered into mice by a hydrodynamic-based gene transfer technique via rapid injection of a large volume of DNA solution through the tail vein, as described elsewhere [62]. Briefly, 20 μg Mcl-1-expressing plasmid (p3×flag-Mcl-1) or control plasmid (p3×flag) was diluted in 1.8 ml of saline and injected by tail vein into the circulation within 5 to 10 s. Three days later, mice were treated with 60 min of warm liver ischemia followed by 6 hr of reperfusion challenge before sacrificed. Part of the liver was processed for TUNEL-based immunohistochemistry and hematoxylin and eosin (HE) staining and the remaining liver was immediately frozen in liquid nitrogen and stored at -80°C for tissue lysate preparation. All animals received human care according to the criteria outlined in the Guide for the Care and Use of Laboratory Animals [63].

### Aminotransferase Levels

About 100 μl of blood was collected from the tail vein. Alanine aminotransferase (ALT) and aspartate aminotransferase (AST) were measured in the Division of Clinical Laboratory of Eastern Hepatobiliary Surgery Hospital by standard procedures.

### HCC Tissue Samples

Liver specimens were obtained from primary HCC patients with HBV infection who received surgical resection in Eastern Hepatobiliary Surgery Hospital of the Second Military Medical University (Shanghai, China) with the approval of The Human Research Committee of University and with the patients' consents.

### Statistical Analysis

Results are expressed as mean ± SD. Statistical evaluation was carried out by one-way analysis of variance (ANOVA) followed by Student-Newman-Keuls test. Correlation between two variables was calculated by Spearman rank correlation coefficient. A value of p < 0.05 was considered to be statistically significant.

## Abbreviations

HBV: hepatitis B virus; HBx: HBV X protein; HCC: human hepatocellular caricinoma; Mcl-1: myeloid cell leukemia-1; PARP: poly (ADP-ribose) polymerase; ROS: reactive oxygen species; GSH: reduced glutathione; GSSG: oxidized glutathione; BHA: butylated hydroxyanisole; hMTH1: human MutT homolog 1; MnSOD: manganese superoxide dismutase; H_2_O_2_: peroxide hydrogen; I/R: ischemia/reperfusion; DHE: dihydroethidine; TUNEL: terminal deoxynucleotidyl transferase dUTP nick end labeling; FACS: fluorescence activated cell sorting; RT-PCR: reverse transcription-polymerase chain reaction; Tg mice: Transgenic mice; WT: wide type; Ad: Adenovirus; M.O.I.: multiplicity of infection; H.E.: hematoxyli-eosin; DMSO: dimethylsulfoxide; SD: standard deviation.

## Competing interests

The authors declare that they have no competing interests.

## Authors' contributions

LH, LC and GZY contributed equally to this work. All authors read and approved the final manuscript.

## Supplementary Material

Additional file 1 **Figure S1 -The expression of flag-Mcl-1 in whole liver extracts**. HBx-Tg mice were subjected to a single injection of Mcl-1-expressing plasmid (p3×flag-Mcl-1) or control plasmid (p3×flag) by tail vein. Two days later, whole liver extracts were probed with ant-Flag antibody. Figure S2 -The expression and mitochondria translocation of Bax/Bak in HBx-expressing cells following H_2_O_2 _treatment. (A) Indicated cells (1 × 10^7^) were treated with or without H_2_O_2 _(400 μM) for 15 hr followed by subjected to cellular fractionation. Whole cell lysates, cytosolic and mitochondrial fractions were subjected to Western blot analysis and probed with anti-Bax or anti-Bak antibody. (Cyto: cytosolic; Mito: mitochondrial). (B) SMMC-7721 cells were transiently transfected with p3.8II or p3.8IIxm for 36 hr followed by treated with H_2_O_2 _(400 μM) for additional 15 hr. Then whole cell lysates, cytosolic and mitochondrial fractions were subjected to Western blot assay and probed with indicated antibodies. (Cyto: cytosolic; Mito: mitochondrial). Click here for file
