# Supplementary figures and images for: HBx Sensitizes Cells to Oxidative Stress-induced Apoptosis by Accelerating the Loss of Mcl-1 Protein via Caspase-3 Cascade
Source: Mol Cancer. 2011 Apr 20;10:43. doi: 10.1186/1476-4598-10-43 (PMC3096594; doi:10.1186/1476-4598-10-43)

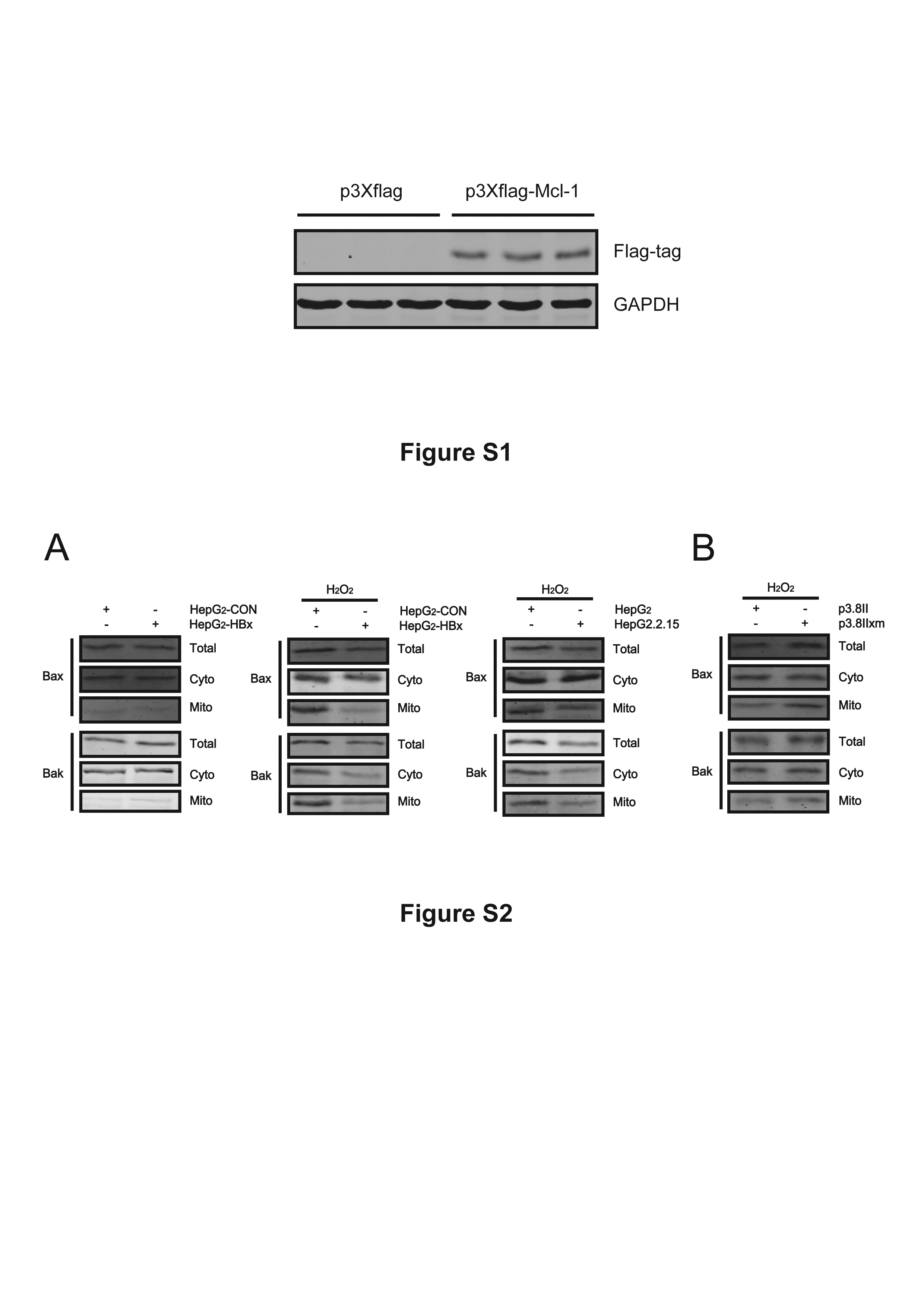

Supplement: Additional file 1 — Figure S1 -The expression of flag-Mcl-1 in whole liver extracts. HBx-Tg mice were subjected to a single injection of Mcl-1-expressing plasmid (p3×flag-Mcl-1) or control plasmid (p3×flag) by tail vein. Two days later, whole liver extracts were probed with ant-Flag antibody. Figure S2 -The expression and mitochondria translocation of Bax/Bak in HBx-expressing cells following H2O2 treatment. (A) Indicated cells (1 × 10^7^) were treated with or without H2O2 (400 μM) for 15 hr followed by subjected to cellular fractionation. Whole cell lysates, cytosolic and mitochondrial fractions were subjected to Western blot analysis and probed with anti-Bax or anti-Bak antibody. (Cyto: cytosolic; Mito: mitochondrial). (B) SMMC-7721 cells were transiently transfected with p3.8II or p3.8IIxm for 36 hr followed by treated with H2O2 (400 μM) for additional 15 hr. Then whole cell lysates, cytosolic and mitochondrial fractions were subjected to Western blot assay and probed with indicated antibodies. (Cyto: cytosolic; Mito: mitochondrial). [file 1476-4598-10-43-S1.JPEG]
